# Gene Expression Profiling of Peri-Implant Healing of PLGA-Li^+^ Implants Suggests an Activated Wnt Signaling Pathway *In Vivo*

**DOI:** 10.1371/journal.pone.0102597

**Published:** 2014-07-21

**Authors:** Anna Thorfve, Anna Bergstrand, Karin Ekström, Anders Lindahl, Peter Thomsen, Anette Larsson, Pentti Tengvall

**Affiliations:** 1 Department of Biomaterials, Institute of Clinical Sciences, The Sahlgrenska Academy, University of Gothenburg, Gothenburg, Sweden; 2 BIOMATCELL VINN Excellence Center of Biomaterials and Cell Therapy, Gothenburg, Sweden; 3 Department of Chemical and Biological Engineering, Chalmers University of Technology, Gothenburg, Sweden; 4 SuMo BIOMATERIALS VINN Excellence Center, Gothenburg, Sweden; 5 Stiftelsen Chalmers Industriteknik, Chalmers Teknikpark, Gothenburg, Sweden; 6 Department of Clinical Chemistry and Transfusion Medicine, Institute of Biomedicine, The Sahlgrenska Academy, University of Gothenburg, Gothenburg, Sweden; Georgia Regents University, United States of America

## Abstract

Bone development and regeneration is associated with the Wnt signaling pathway that, according to literature, can be modulated by lithium ions (Li^+^). The aim of this study was to evaluate the gene expression profile during peri-implant healing of poly(lactic-co-glycolic acid) (PLGA) implants with incorporated Li^+^, while PLGA without Li^+^ was used as control, and a special attention was then paid to the Wnt signaling pathway. The implants were inserted in rat tibia for 7 or 28 days and the gene expression profile was investigated using a genome-wide microarray analysis. The results were verified by qPCR and immunohistochemistry. Histomorphometry was used to evaluate the possible effect of Li^+^ on bone regeneration. The microarray analysis revealed a large number of significantly differentially regulated genes over time within the two implant groups. The Wnt signaling pathway was significantly affected by Li^+^, with approximately 34% of all Wnt-related markers regulated over time, compared to 22% for non-Li^+^ containing (control; Ctrl) implants. Functional cluster analysis indicated skeletal system morphogenesis, cartilage development and condensation as related to Li^+^. The downstream Wnt target gene, *FOSL1*, and the extracellular protein-encoding gene, *ASPN*, were significantly upregulated by Li^+^ compared with Ctrl. The presence of β-catenin, FOSL1 and ASPN positive cells was confirmed around implants of both groups. Interestingly, a significantly reduced bone area was observed over time around both implant groups. The presence of periostin and calcitonin receptor-positive cells was observed at both time points. This study is to the best of the authors' knowledge the first report evaluating the effect of a local release of Li^+^ from PLGA at the fracture site. The present study shows that during the current time frame and with the present dose of Li^+^ in PLGA implants, Li^+^ is not an enhancer of early bone growth, although it affects the Wnt signaling pathway.

## Introduction

Orthopedic and dental implant therapies have evolved into important treatments for deranged joints and lost teeth or to provide fixation of bone in the case of fractures. Osteogenesis, i.e. the differentiation of mesenchymal stem cells (MSCs) into mature osteoblasts is essential in bone growth, fracture healing and osseointegration. The hallmarks of osteogenesis around implants are increased alkaline phosphatase (ALP) activity and the formation of a calcium-rich mineralized extracellular matrix (ECM). This contains bone-related proteins, such as type I collagen (COL1), osteocalcin (OCN), bone sialoprotein (BSP) and osteopontin (OPN)[1]. The runt-related transcription factor 2 (RUNX2) is indicated as the master switch in osteogenesis, although other factors, such as the canonical Wnt signaling pathway are pivotal for the guidance of MSCs into the osteoblastic lineage and bone homeostasis[2], [3]. The canonical Wnt signaling pathway involves several Wnt proteins that, upon activation, bind to receptors frizzled (FZD) and co-receptor LDL-related proteins 5/6 (LRP5/6). This interaction initiates a signaling cascade that leads to the inactivation of the β-catenin (CTNNB1) degradation complex, consisting of AXIN, adenomatous polyposis coli (APC), casein kinase I (CK1) and glycogen synthase kinase 3b (GSK-3β). The intracellular β-catenin concentrations then become stabilized, translocated into the nucleus and activate the transcription of downstream canonical Wnt-related genes. The non-canonical pathways, which function independently of β-catenin, are less well studied but attract increasing interest. Recent studies suggests the FZD9 receptor as a positive regulator of both intramembranous and endochondral ossification during fracture healing via the non-canonical pathways[4], [5], although the canonical Wnt signaling pathway is implicated as the dominant mechanism in bone biology. The critical role of Wnt signaling is well recognized, not only during embryonic bone formation, post-natal bone homeostasis and regeneration but also for osseointegration of implants[6]–[8]. Of several ways to modulate the Wnt signaling pathway, lithium ions (Li^+^) are often regarded as the simplest activator[9], [10]. Oral Li^+^ treatment, widely used as stabilizer in bipolar and depressive disorders, is reported to activate the canonical Wnt signaling pathway via the inhibition of the β-catenin degradation enzyme, GSK-3β[11], [12]. Previous studies indicated that Li^+^ induces increased bone formation and bone mass in mice, as well as reduce the risk of fractures in patients on Li^+^ treatment[13], [14]. However, due to the fact that Li^+^ has a narrow therapeutic index and the therapy is associated with multiple side-effects[11], it would be beneficial in biomaterial applications to enhance bone regeneration via a local release of Li^+^ from bioactive degradable materials. The objective of the present study was to incorporate Li^+^ into poly(lactic-co-glycolic acid) (PLGA) implants, to monitor the local release of Li^+^ and to evaluate the local biological effects by gene expression, immunohistochemistry, histology and histomorphometry in a rat tibia model. PLGA is a biodegradable co-polymer that is widely used in pharmaceutics as a controlled drug delivery system[15] and it has evolved into a frequently used synthetic polymer within the field of bone regeneration[16]. One reason for its widespread use is its biodegradability and biocompatibility. In the presence of water, PLGA degrades via hydrolysis into lactic and glycolic acids, natural compounds that are metabolized and excreted as carbon dioxide and water[16], [17]. PLGA is in clinical use since decades and its biosafety is proven in many medical applications[16], [18]. It has been used as a carrier for the delivery of osteogenic factors for cell adhesion, differentiation and improved bone regeneration, as pure PLGA implants or in combination with hydroxyapatite (HA) to obtain an improved mechanical strength[19]–[23]. To study the effect of Li^+^, the ions have previously been incorporated into various materials or added directly to cell culture media[24]–[27]. However, there is no previous study using Li^+^-PLGA implants with the aim to modulate the Wnt signaling pathway *in vivo*. In order to fully investigate the underlying cellular and molecular mechanisms of peri-implant healing within this context, we performed a genome-wide microarray analysis, followed by validation of selected results by qPCR, in combination with a histomorphometric evaluation. Previous works used gene expression profiling during *in vivo* bone healing, with or without implants[28]–[32], but this is to the best of the authors' knowledge, the first *in vivo* study to evaluate the bone healing aspects in the vicinity of Li^+^-containing PLGA implants. We were able to show that the present dose of Li^+^ activates the Wnt signaling pathway but is not an inducer of early bone growth. In addition to providing insights into Wnt signaling during peri-implant healing around bone-anchored implants, this study shows that a local release of Li^+^ at the fracture site can be used to modulate bone cell signaling but needs further optimization in order to induce early bone growth.

## Materials and Methods

### Li^+^-PLGA implant fabrication, characterization and Li^+^ release profile

Lithium salt containing plug-shaped samples and control samples made of sodium salt were prepared as follows. 10 g of lithium carbonate (Sigma Aldrich, St. Louis, MO, USA) or sodium carbonate salts (Sigma Aldrich) were ground manually with a mortar and pestle. The ground powder was transferred to and sieved through a set of sieves with sizes 45–180 µm at maximum amplitude for 5 minutes using a vibratory sieve shaker (Rietsch, Germany). A batch of 10 g lithium carbonate or sodium carbonate salt generated a 45–90 µm sieve fraction of about 2 g salt. The ground and sieved salts were mixed with 50∶50 PLGA powder, MW 24,000–38,000 (Sigma Aldrich) at a 1∶10 ratio (w/w), hot-melt pressed at 100°C and pre-pelletized. The PLGA pellets with included salt were repeatedly fed into a HAAKE MiniLab rheometer (Thermo Scientific, Rockford, IL, USA), at 100°C, speed 60 l/min, and extruded through a die with a diameter of 1.6 mm. The strains were cut and heat-molded to form a plug with dimensions: d_head_ = 3.5 mm, d_shank_ = 1.8–2.0 mm, l_tot_ = 3.2 mm, h = 1.0 mm. The weight of a plug was typically 17 mg. The salt containing PLGA sample plugs will henceforth be designated Li^+^ and Ctrl implant, respectively.

### Scanning Electron Microscopy

The internal pore structure, morphology and porosity of the cross-sectioned implants were characterized by a field emission scanning electron microscope (SEM, Leo Ultra 55 FEG SEM, Leo Electron Microscopy Ltd, Cambridge, UK) at 3 kV. The implants that had undergone ion release experiments (day 0–28; see below) were embedded in plastic resin (LR White; Sigma-Aldrich) and polymerized prior to cutting along the long axis of the implant, sputter-coated with gold before analysis and visualized using the in-lens detection mode.

### TOF-SIMS Imaging

The chemical characterization of the implants was performed with time-of-flight secondary ion mass spectrometry (TOF-SIMS, TOF-SIMS IV, ION-TOF GmbH, Münster, Germany), using a primary ion beam of 25 keV Bi^3+^ ions. The specimens were embedded in plastic resin (as described above). A thin section was prepared by cutting and grinding to achieve a final thickness of 10–20 µm and the samples were cleaned in N_2_ gas before analysis. In order to evaluate the Li^+^ distribution in the implant during *in vitro* degradation, stage scan imaging of positive ions was performed in the bunched mode (PI target current 0.1 pA).

### Li^+^ release *in vitro*

For Li^+^ release profile analysis, PLGA implants with included lithium carbonate salt were submerged in 10 mL PBS buffer, pH 7.2, and agitated at 37°C for 4 weeks on a rotating table. Samples of 1 mL were collected at specified time points and replaced by 1 mL PBS buffer. Li^+^ levels were well below sink conditions throughout the experiment. The amount of released Li^+^ was determined using flame emission spectrometry (iCE 3300 AA Spectrometer, ThermoScientific, Germany), with a detection wavelength of 670.8 nm.

### Ethics Statement

The research described in this study involving animal experiments was approved by the University of Gothenburg's Local Ethical Committee for Laboratory Animals (Dnr: 279–2011).

### Surgical procedure

The implants were sterilized by ultra violet (UV) treatment for 1 hour and endotoxin analysis was performed with *Limulus amebocyte* lysate using a kinetic chromogenic method (Charles River, L′arbresle Cedex, France), and run according to the FDA protocol. All implants showed values below the recommended maximum level of 1.25 endotoxin units (EU) per rat (250 g) (Sahlgrenska Hospital, Gothenburg, Sweden). Thirty-four male Sprague-Dawley rats (390–400 g), fed on a standard pellet diet and water, were anesthetized using a Univentor 400 anesthesia unit (Univentor, Zejtun, Malta) under isoflurane (Isoba Vet, Schering-Plough Uxbridge, England) inhalation (4% with an air flow of 650 mL/min). Anesthesia was maintained by the continuous administration of isoflurane (2.7% with an air flow of 450 mL/min) via a mask, and all efforts were made to minimize suffering. Each rat received analgesic (Temgesic 0.03 mg/kg, Reckitt & Coleman, Hull, Great Britain) subcutaneously prior to implantation and every day postoperatively. After shaving and cleaning (5 mg/mL chlorhexidine in 70% ethanol), the medial aspect of the proximal tibial metaphysis was exposed through an anteromedial skin incision, followed by skin and periosteum reflection with a blunt instrument. After bone preparation with 1.8- and 2.1-mm burrs under profuse irrigation with NaCl 0.9%, 2 implants (one from each implant group) were inserted manually in each rat tibia. The locations of implants were decided using a predetermined schedule, ensuring alteration between the legs and sites. The subcutaneous layer of the wound was closed with resorbable polyglactin sutures (5-0, Vicryl, Ethicon, Johnson & Johnson, Brussels, Belgium) and the skin was closed with transcutaneously placed non-resorbable nylon sutures (4-0, Ethilon, Ethicon, Johnson & Johnson). The animals were allowed free postoperative movement, with food and water *ad libitum*. The retrieval procedure was performed at 7 and 28 days (17 rats at each time point). The rats were sacrificed using an intraperitoneal overdose of sodium pentobarbital (60 mg/mL; ATL Apoteket Production & Laboratories, Sweden) under anesthesia, with a 0.5 mL mixture of pentobarbital (60 mg/mL), sodium chloride and diazepam (1∶1∶2) and cleaned with 5 mg/mL of chlorhexidine in 70% ethanol. After cutting the sutures, an incision of the skin and a reflection of the periosteum were performed. For transcript profiling analyses, peri-implant bone was carefully collected using a 3.8 mm diameter trephine burr and immediately placed in RNA preservation solution and stored at 4°C until analysis. In addition, bone-implant blocks were fixated for subsequent microscopic examination.

### RNA isolation

Bone samples were homogenized in a phenol/guanidine-based Qiazol lysis reagent, using 5-mm stainless steel beads (QIAGEN GmbH, Hilden, Germany) and TissueLyser (QIAGEN). Following the addition of chloroform, the samples were centrifuged at 12,000 g for 15 min and the aqueous phase was used for subsequent RNA extraction. Total RNA from the surrounding bone was extracted using a RNeasy Mini kit (QIAGEN), according to the manufacturer's instructions. DNAse treatment was performed in order to eliminate any contamination from genomic DNA. The quantification and quality assessment of RNA was performed using the Agilent Bioanalyzer (Agilent Technologies, Santa Clara, CA, USA) and NanoDrop spectrophotometer (Thermo Scientific, Rockford, IL, USA). All samples were of similar RNA quality and integrity (RIN>8).

### Global transcriptional and microarray data analysis

Total RNA (200 ng) from 12 peri-implant bone samples/time point (n = 6 Li^+^; 6 = Ctrl) was subjected to gene expression analysis using the Affymetrix Rat Gene 2.0 ST Array (Affymetrix, Santa Clara, CA, USA), and handled according to the manufacturer's recommendations. Expression data were normalized and summarized using the RMA algorithm implemented in the Affymetrix Expression Console version 1.1.2 software[33]. T-test analyses (paired; Li^+^ vs. Ctrl and unpaired; Li^+^ day 28 vs. 7, Ctrl day 28 vs. 7) were performed on log2-transformed signal values to identify significantly differentially expressed genes between groups, using the TMEV v4.0 software. Expression differences were given as fold changes (FCs); only significantly altered genes that displayed a mean fold change of FC≥1.5 or ≤−1.5 (p<0.05) were selected for further analysis. To further investigate the biology of the differentially regulated genes, the DAVID software tool[34], [35] (http://david.abcc.ncifcrf.gov/) was used to explore their gene ontology (GO) annotations according to biological processes. To identify significantly differentially regulated pathways and specific Wnt signaling-related genes during the peri-implant healing processes, the KEGG pathway database (http://www.genome.jp/kegg/) was used. All significantly regulated genes (p<0.05) obtained from the microarray analysis (no specific FC limit) were included in the gene list. The microarray data discussed in this publication followed MIAME requirements and have been deposited in NCBI's Gene Expression Omnibus (GEO)[36] with the GEO Series accession number GSE54294.

### Quantitative real-time PCR (qPCR) analysis

Briefly, RNA was transcribed to cDNA using a HighCapacity cDNA Reverse Transcription Kit (Life Technologies, Carlsbad, USA), according to the manufacturer's instructions. The instrument, 7900 HT, software and reagents for the real-time qPCR analysis were purchased from Life Technologies. The commercially available assay-on-demand mixes of primers and TaqMan MGB probes (FAM dye-labeled) were used in this study: *ACAN* (Rn00573424_m1), *ASPN* (Rn01757407_m1), *A2M* (Rn00560589_m1), *CXCL2* (Rn00586403_m1), *DKK1* (Rn01501537_m1), *FOSL1* (Rn00564119_m1), *FZD2* (Rn00597004_s1), *LRP5* (Rn01451428_m1), *PRKCA* (Rn01496145_m1), *PRSS34* (Rn01466862_g1) and *SFRP5* (Rn01766277_m1). *18s* (Hs9999901_s1) was used as an endogenous control. cDNA corresponding to 2.5 ng total RNA were analyzed in duplicate for all samples (n = 8). Relative gene expression level (the amount of target, normalized to the endogenous control gene) was calculated using the 2^−ΔΔCt^ method[37] in GenEx Enterprise 5.2.3.13 (MultiD analyses, Gothenburg, Sweden) and expressed as relative quantities normalized to the Ctrl group at 7 days post-surgery, with a given reference value 1.

### Histology and Immunohistochemistry

For histological evaluations, the bone-implant specimens were harvested, PLGA remnants were removed from the defect site and the tissue blocs were fixated in formalin, dehydrated and embedded in paraffin and sectioned (3–5 µm). The sections were subsequently mounted on StarFrost slides (Waldemar Knittel Glasbearbeitungs-GMbH, Braunschweig, Germay) and maintained at 60°C in an oven for 1 h. The sections were either stained with hematoxylin/eosin (HE; Merck KGaA, Darmstadt, Germany) for histological evaluation or processed for immunohistochemical (IHC) staining; the sections were deparaffinized in xylene, hydrated in descending series of ethanol and treated with 3% H_2_O_2_, and antigen retrieval with 10 mM citrate buffer was performed. Slides were blocked with 3% BSA for non-specific antibody binding before incubation with either of primary antibodies from Abcam (Abcam, Cambridge, MA, USA); β-catenin (ab16051), FOSL1 (ab117951), ASPN (ab58741), periostin (ab14041) and calcitonin receptor (ab11042). As negative controls, some tissue samples were incubated without the primary antibody or with isotype controls (data not shown). The primary antibody was visualized using HRP-conjugated secondary antibodies and a Betazoid DAB (3,30-diaminobenzidine) Chromogen kit (Biocare Medical, Concod, CA, USA). All slides were counterstained with Mayer's hematoxylin (Merck KGaA) and examined with light microscopy (Nikon Eclipse E600).

### Histomorphometry

HE-stained sections were used for a blinded quantitative histomorphometric analysis by measuring the bone area (BA) around the implantation site. The BA was measured in the square area extending 500 µm from the implantation/defect site into bone. In order to capture all areas dominated by newly formed bone, the analysis was performed on two separate compartments – Part A (longitudinally with the plug) and Part B (underneath the plug). The analysis was performed at 10× magnification and all specimens were evaluated using light microscopy (Nikon Eclipse E600, Japan).

### Statistics

The statistical analysis was performed with GenEx (MultiD analyses) and SPSS v19 (IBM Corp., Armonk, NY, USA) software. Logarithmic values of the gene-expression data were used for statistical calculations. Statistical significance was determined using Student's t-test (paired; Li^+^ vs. Ctrl and unpaired; Li^+^ day 28 vs. 7, Ctrl day 28 vs. 7). When the normal distribution of the data could not be guaranteed, equivalent non-parametric tests were used. A significant difference was assumed at a p-value of ≤0.05. Unless otherwise stated, the data are expressed as the mean ± standard deviations.

## Results

### *In vitro* characterization of PLGA implants

**Figure 1:**
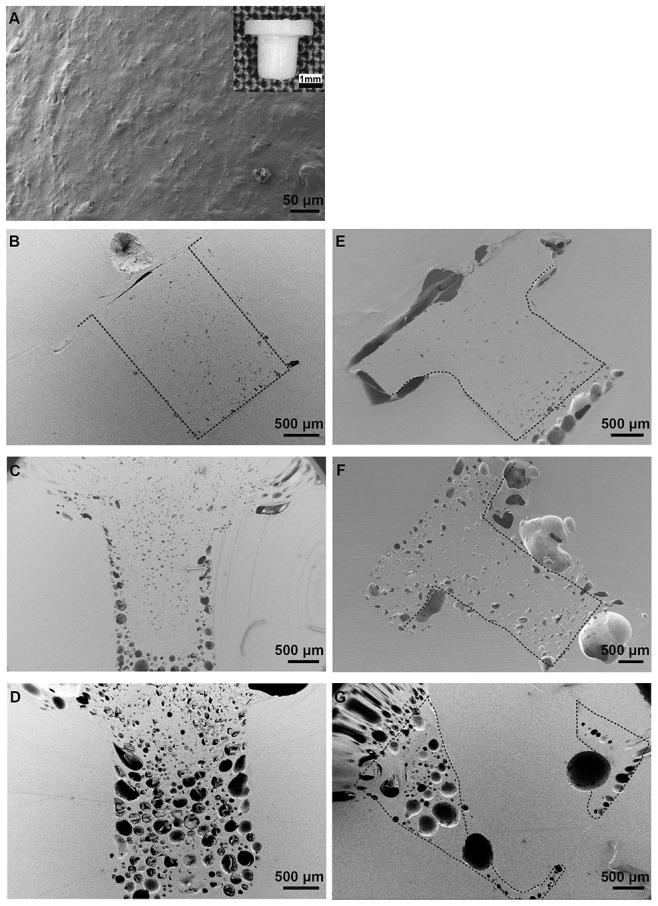
Scanning electron micrographs of PLGA implant cross-sections. SEM images showing surface and cross-sections of PLGA implants exposed to PBS buffer for 0, 7 and 28 days. **A.** Surface of Li^+^- or Na^+^-containing (Ctrl) implants, day 0 and **B.** cross-section of Li^+^-containing and **E.** Ctrl implant not exposed to buffer, day 0. **C.** Cross-sections of Li^+^-containing and **F.** Ctrl implant after submersion (day 7) or **D.** Li^+^-containing and **G.** Ctrl implant day 28 in PBS buffer. Design and surface of plug-shaped PLGA implant, see insert **A**. Implant dimensions; d_head_ = 3.5 mm, d_shank_ = 1.8–2.0 mm, l_tot_ = 3.2 mm, h = 1.0 mm. Pores and air bubbles created during sample preparation for microscopy analysis appeared as black circles in images and were mainly found between the implant and embedding material. The hatched line defines the shape of the plug.

**Figure 2:**
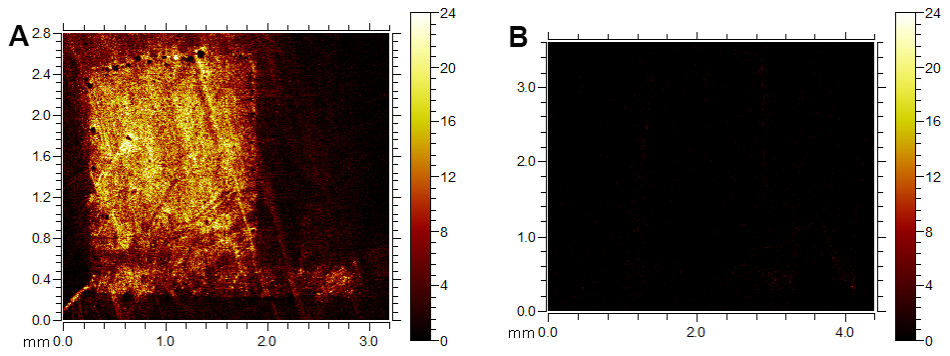
TOF-SIMS ion imaging stage scans of PLGA implants. Images show the Li^+^ intensity (the sum of intensities for the isotopes ^6^Li and ^7^Li) of **A.** Li^+^-containing PLGA implant and **B.** Ctrl implant without Li^+^, at day 0.

**Figure 3:**
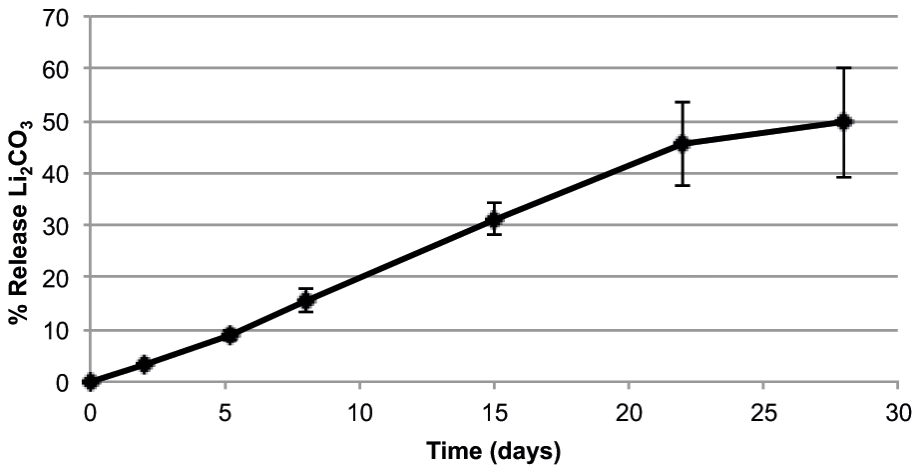
*In vitro* Li^+^ release profile in PBS. Percentage (%) release of Li_2_CO_3_ from PLGA implants (error bars represent the standard deviations, n = 3).

The aim with incorporation of Li^+^ into PLGA implants was to obtain a locally controlled release of Li^+^ out to the surrounding bone. The *in vitro* implant characterization was performed in order to follow eventual changes in implant morphology upon the Li^+^ release to buffer. The implant surfaces displayed initially a smooth surface with few irregularities and limited presence of visible pores (Fig. 1A). A dense morphology with some irregularities, small pores and cavities were observed at the surface of cross-sectioned, non-submerged Li^+^- and Na^+^-containing (Ctrl) implants (day 0) (Fig. 1B and E, respectively). Larger pores were observed at the bottom part of the Ctrl implant (Fig. 1E), although these were judged as artifacts created during sample preparation. The cross-sections of Li^+^-containing samples submerged for 7 or 28 days in buffer showed large cavities at the outer part of the shaft (Fig. 1C and D, respectively). A similar morphology was observed for the Ctrl samples, although with less porosity after 7 days compared to Li^+^ specimens (Fig. 1F and G, respectively). The cavities on Li^+^-containing implants after 7 days of immersion in PBS were mainly located at the outer part of the shaft, but after 28 days both types of implants showed cavities also in the central part. Due to artifacts created during sample preparation for microscopy analysis, only fragments of Ctrl implant submerged for 28 days could be visualized by SEM, see Figure 1G. The sizes of the cavities increased over time for both implant types. The TOF-SIMS analysis showed the Li^+^ content in Li^+^-containing implants (Fig. 2A) compared to Ctrl implants (Fig. 2B) at day 0. Pores and air bubbles appeared as black circles in the image. Air bubbles were trapped during sample preparation and were mainly found between the implant and the embedding material. The results confirmed the appearance in SEM images, demonstrating increasing porosity with time, starting from the outside and protruding inwards in the shaft region. The total ion content intensity decreased slightly over time, but remained over the complete polymer matrix part of the implant. The Li^+^ ion intensity was, however, most intense at the inner part of the implant shaft (data not shown). Figure 3 shows Li^+^ release from PLGA implants during 0–28 days in PBS buffer, demonstrating a sustained, almost linear release of Li^+^, reaching about 50% of total Li^+^ content at 28 days. (The results further show a tendency towards a slightly lower release rate after 22 days.)

### Microarray analysis

In order to explore the regulatory mechanisms by which the incorporated Li^+^ affected the peri-implant healing, a genome-wide microarray analysis of the peri-implant bone was performed at 7 and 28 days postoperatively, see Table 1. A total of 1,042 genes were differentially expressed at day 7 of Li^+^ implant vs. Ctrl and 1,348 genes at the later time point (Tables S1–S2). The over-time analysis of each implant group showed 3,359 genes differentially regulated in the control group (Ctrl day 28 vs. 7), and 4,752 genes in the Li^+^ group (Li^+^ day 28 vs. 7) (Tables S3–S4). In-depth search for genes fulfilling the search criteria (FC≥1.5 or ≤−1.5) revealed only a few genes (3–5) as differentially regulated by Li^+^ compared with Ctrl (Tables S1–S2). Over-time analyses revealed 311 differentially regulated genes in the control group; 69 upregulated and 242 downregulated (Table S3). The Li^+^ group showed 342 regulated genes; 111 upregulated and 231 downregulated (Table S4).

**Table 1:** Differentially regulated genes.

Numbers of differentially regulated genes (p<0.05, no specific FC limit)						
	Total		Increase		Decrease	
Li+ day 7 vs. Ctrl	1042		427		615	
Li+ day 28 vs. Ctrl	1348		684		664	
Ctrl day 28 vs. 7	3359		1465		1894	
Li+ day 28 vs. 7	4752		2439		2313	
Numbers of differentially regulated genes (p<0.05, FC≥1.5 or ≤−1.5)						
	Total		Increase		Decrease	
Li+ day 7 vs. Ctrl	3		1		2	
Li+ day 28 vs. Ctrl	5		1		4	
Ctrl day 28 vs. 7	311		69		242	
Li+ day 28 vs. 7	342		111		231	

For complete lists, refer to Tables S1–S4.

### Functional cluster analysis

A functional cluster analysis based on the GO category biological processes was carried out on genes that fulfilled the search criteria. For the Li^+^-containing specimens, the analysis showed 29 downregulated clusters over time; 10 clusters with the highest enrichment scores are listed in Table S5. These were related to extracellular matrix organization, inflammatory response, cell migration, blood vessel development and skeletal system, and cartilage development and condensation. For the Li^+^ group, 2 annotation clusters were upregulated over time; wound healing and regulation of cell activation (Table S6). For the Ctrl group, 25 annotation clusters were downregulated over time; 10 clusters with the highest enrichment scores are listed in Supporting information Table S7. The clusters were related to extracellular matrix organization, inflammatory response, cell migration, blood vessel development and skeletal system, bone development and ossification. Only 1 annotation cluster related to the regulation of cell activation was upregulated (Table S8).

### Clustering of inflammation associated genes

The above mentioned functional cluster analysis demonstrated a slight change in enrichment score between Li^+^ and Ctrl implants with respect to inflammation. The Li^+^ group revealed clusters related to the terms “Inflammatory response” and “Defense response” with an enrichment score of 2.83 (cluster #6, Table S5). The Ctrl group also demonstrated clusters associated to these specific clusters, but additionally also related to the term “Acute inflammatory response” (see cluster #3, Table S7), and showed a higher enrichment score; 3.67. Some of the most differentially expressed genes within these clusters were *A2M* (FC - 4.01 Li^+^; FC - 3.19 Ctrl), *CCL7* (FC - 3.64 Li^+^; FC - 2.72 Ctrl), and *CXCL2* (FC - 4.76 Ctrl), see Tables 2 and 3.

**Table 2:** Inflammation associated genes downregulated in Li^+^ day 28 vs. 7.

Probe Set ID	Gene Accession	Gene Symbol	Gene Description	FC Li+ day 28 vs 7	P-value
17786724	NM_012488	A2M	Alpha-2-macroglobulin	−4.01	3.10E-05
17649432	NM_001007612	CCL7	Chemokine (C-C motif) ligand 7	−3.64	5.32E-04
17649426	NM_031530	CCL2	Chemokine (C-C motif) ligand 2	−2.60	9.23E-04
17711968	ENSRNOT00000014809	SLC7A2	Solute carrier family 7 (cationic amino acid transporter, y+ system), member 2	−2.15	2.76E-04
17812245	ENSRNOT00000020316	PDPN	Podoplanin	−1.95	0.002
17659402	NM_013025	CCL3	Chemokine (C-C motif) ligand 3	−1.81	0.025
17680795	ENSRNOT00000003567	PTGS2	Prostaglandin-endoperoxide synthase 2	−1.79	0.043
17742303	NM_013057	F3	Coagulation factor III (thromboplastin, tissue factor)	−1.79	0.006
17777192	NM_031512	IL1B	Interleukin 1 beta	−1.72	0.045
17667363	ENSRNOT00000040065	EPHA3	Eph receptor A3	−1.72	0.005
17718447	XM_225216	S1PR3	Sphingosine-1-phosphate receptor 3	−1.67	0.005
17637979	NM_053420	BNIP3	BCL2/adenovirus E1B interacting protein 3	−1.62	0.017
17759790	NM_022194	IL1RN	Interleukin 1 receptor antagonist	−1.60	0.002
17795409	NM_133306	OLR1	Oxidized low density lipoprotein (lectin-like) receptor 1	−1.56	0.015

Data derived from the annotation cluster #6 in Table S5.

**Table 3:** Inflammation associated genes downregulated in Ctrl day 28 vs. 7.

Probe Set ID	Gene Accession	Gene Symbol	Gene Description	FC Ctrl day 28 vs 7	P-value
17693459	ENSRNOT00000003745	CXCL2	Chemokine (C-X-C motif) ligand 2	−4.76	0.047
17786724	NM_012488	A2M	Alpha-2-macroglobulin	−3.19	2.15E-04
17649432	NM_001007612	CCL7	Chemokine (C-C motif) ligand 7	−2.72	0.002
17676856	ENSRNOT00000001916	SERPINE1	Serpin peptidase inhibitor, clade E (nexin, plasminogen activator inhibitor type 1), member 1	−2.54	7.63E-04
17649426	NM_031530	CCL2	Chemokine (C-C motif) ligand 2	−2.38	0.002
17659402	NM_013025	CCL3	Chemokine (C-C motif) ligand 3	−2.35	1.22E-04
17726248	NM_017061	LOX	Lysyl oxidase	−2.18	0.018
17610985	NM_017134	ARG1	Arginase, liver	−2.17	0.002
17711968	ENSRNOT00000014809	SLC7A2	Solute carrier family 7 (cationic amino acid transporter, y+ system), member 2	−2.13	0.005
17812245	ENSRNOT00000020316	PDPN	Podoplanin	−2.08	0.020
17667363	ENSRNOT00000040065	EPHA3	Eph receptor A3	−1.98	0.011
17687457	ENSRNOT00000003313	TGFB2	Transforming growth factor, beta 2	−1.98	0.009
17747609	ENSRNOT00000018628	IGSF10	Immunoglobulin superfamily, member 10	−1.96	0.026
17759790	NM_022194	IL1RN	Interleukin 1 receptor antagonist	−1.85	0.001
17672282	NM_012801	PDGFA	Platelet-derived growth factor alpha polypeptide	−1.84	0.008
17795409	NM_133306	OLR1	Oxidized low density lipoprotein (lectin-like) receptor 1	−1.79	0.024
17630332	ENSRNOT00000041891	APOE	Apolipoprotein E	−1.72	0.007
17625032	NM_001106375	PAPSS2	3′-phosphoadenosine 5′-phosphosulfate synthase 2	−1.72	0.022
17725368	ENSRNOT00000027739	NREP	Neuronal regeneration related protein	−1.69	0.023
17858944	NM_032085	COL3A1	Collagen, type III, alpha 1	−1.67	0.030
17746926	ENSRNOT00000019554	ANXA5	Annexin A5	−1.62	0.002
17731914	NM_031054	MMP2	Matrix metallopeptidase 2	−1.58	0.033
17742303	NM_013057	F3	Coagulation factor III (thromboplastin, tissue factor)	−1.57	0.003
17734841	NM_017091	PCSK1	Proprotein convertase subtilisin/kexin type 1	−1.56	0.007
17718447	XM_225216	S1PR3	Sphingosine-1-phosphate receptor 3	−1.54	0.018
17719775	NM_080405	GLI3	GLI family zinc finger 3	−1.54	0.041
17789522	ENSRNOT00000013989	TFPI2	Tissue factor pathway inhibitor 2	−1.52	0.041

Data derived from the annotation cluster #3 in Table S7.

### Pathway analysis and differentially expressed Wnt-related markers

In order to explore the pathways that could be differentially expressed over time in both implant groups, the KEGG pathway database was searched with the genes differentially expressed as input. For the Li^+^ specimens, 9 pathways were downregulated at day 28 compared with 7 days post-surgery (Table 4). The KEGG term “Pathways in cancer” (Rno:05200) was one of them; this term includes the Wnt signaling pathway (Rno:04310). Only 2 pathways were shown to be upregulated on day 28 compared with day 7 in the Li^+^ group, see Table 5. For the Ctrl group 6 pathways with decreased expression 28 days post-surgery were revealed (Table 6). No pathways were significantly upregulated over time. The presence of the Wnt signaling pathway in the Li^+^ group but not in the Ctrl demonstrates that the Wnt signaling network was regulated over time in the presence of Li^+^. Since this recognized Wnt activator was used in this study, a more specific search was performed on all Wnt-related significantly regulated genes, according to the KEGG database. Four genes were differentially expressed on day 7 in Li^+^ implants vs. Ctrl and, at the later time point, 9 genes. The analysis revealed 34 Wnt-related genes differentially regulated over time within the Li^+^ group and 22 genes in the Ctrl group (Table 7 and Tables S9–12). Interestingly, the negative Wnt regulator, β-catenin interaction protein 1 (*CTNNBIP1*), was downregulated at 7 days after implantation in the Li^+^ group (FC -1.12) (Table S9). Moreover, β-catenin (*CTNNB1*) was downregulated at 28 days post-surgery in the presence of Li^+^ (FC -1.21), but was not affected in the Ctrl group (Tables S11–12).

**Table 4:** KEGG pathways with genes downregulated in Li^+^ day 28 vs. 7.

KEGG ID	Term	Count	P-value
rno04360	Axon guidance	9	1.49E-04
rno04510	Focal adhesion	10	5.88E-04
rno04512	ECM-receptor interaction	6	0.003
rno00330	Arginine and proline metabolism	5	0.004
rno05211	Renal cell carcinoma	5	0.010
rno05200	Pathways in cancer	10	0.015
rno05210	Colorectal cancer	5	0.017
rno04060	Cytokine-cytokine receptor interaction	7	0.034
rno04621	NOD-like receptor signaling pathway	4	0.041

Count indicates the total number of genes from the input list that belong to the corresponding term; only significantly expressed genes (no specific FC limit) were included in the gene list.

**Table 5:** KEGG pathways with genes upregulated in Li^+^ day 28 vs. 7.

KEGG ID	Term	Count	P-value
rno04640	Hematopoietic cell lineage	6	1.99E-05
rno04512	ECM-receptor interaction	5	4.11E-04

Count indicates the total number of genes from the input list that belong to the corresponding term; only significantly expressed genes (no specific FC limit) were included in the gene list.

**Table 6:** KEGG pathways with genes downregulated in Ctrl day 28 vs. 7.

KEGG ID	Term	Count	P-value
rno04510	Focal adhesion	12	4.87E-05
rno04512	ECM-receptor interaction	8	9.27E-05
rno00330	Arginine and proline metabolism	6	6.60E-04
rno04360	Axon guidance	7	0.006
rno04062	Chemokine signaling pathway	7	0.026
rno04621	NOD-like receptor signaling pathway	4	0.049

Count indicates the total number of genes from the input list that belong to the corresponding term; only significantly expressed genes (no specific FC limit) were included in the gene list.

**Table 7:** Number of differentially regulated Wnt genes.

	Total	Increase	Decrease
Li+ day 7 vs. Ctrl	4	1	3
Li+ day 28 vs. Ctrl	9	4	5
Ctrl day 28 vs. 7	22	7	15
Li+ day 28 vs. 7	34	12	22

P<0.05, no specific FC limit. For complete lists, refer to tables S9–S12.

### qPCR validation of microarray data

**Figure 4:**
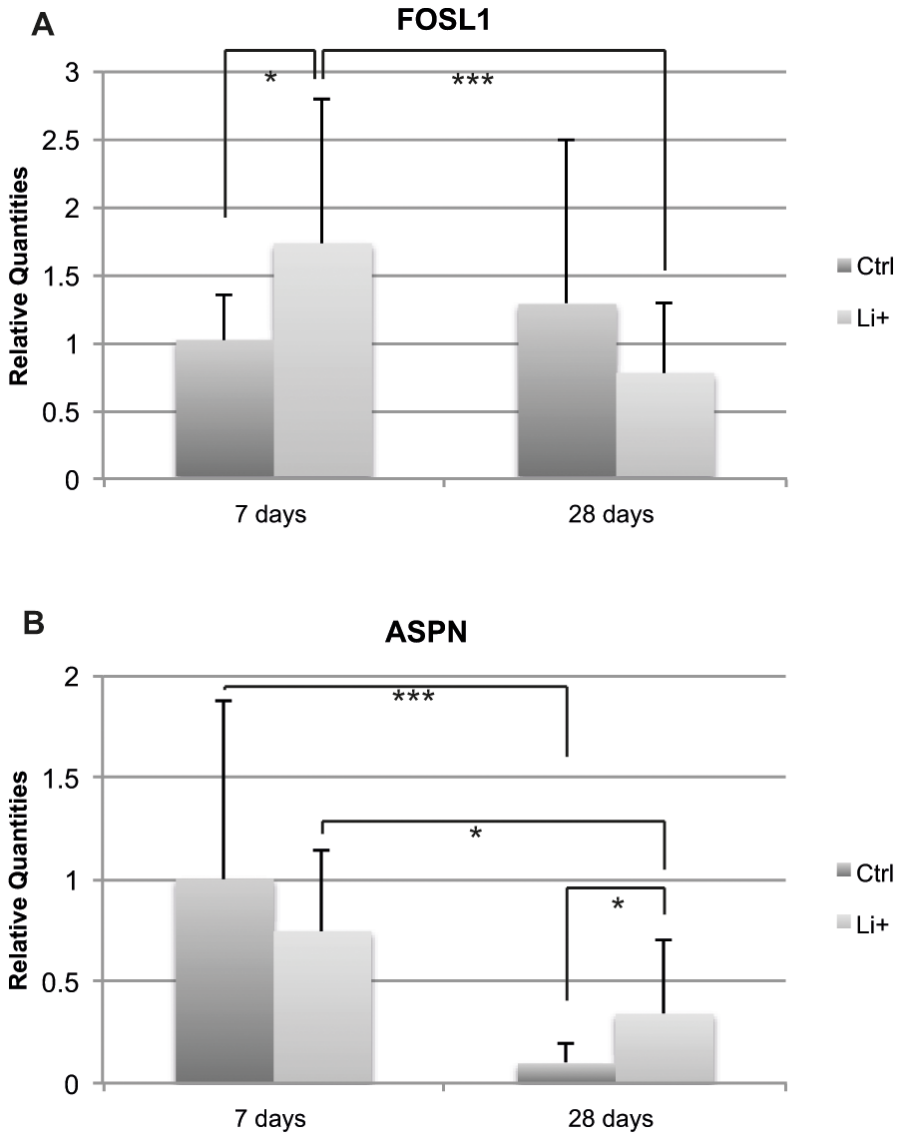
Gene expression analysis of FOSL1 and ASPN in peri-implant bone. Relative gene expression level quantified by qPCR in the peri-implant bone at 7 and 28 days postoperatively for Li^+^ and Ctrl implants. **A.** FOSL1 and **B.** ASPN. Expressed as relative quantities normalized to Ctrl at day 7 post-surgery (reference value 1). A paired Student's t-test or Wilcoxon signed-rank test was used for the Li^+^ vs. Ctrl statistical analysis, whereas unpaired Student's t-test or Mann-Whitney test was used for statistical significance analysis of Li^+^ and Ctrl over time (* p<0.05, ** p<0.01, ***p<0.001).

In order to confirm the gene expression profile from the microarray analysis, the expression of some of the most differentially regulated genes over time from both groups was further analyzed by qPCR (Table 8). The overall FC obtained from the microarray tended to be smaller than that from the qPCR analysis, although the datasets were largely in accordance with each other, verifying the microarray results. In the gene array dataset (Tables S9–10), we observed no major differences between the Li^+^ and Ctrl groups. However, qPCR revealed a difference in gene expression between Li^+^ and Ctrl for the Wnt related genes *FOSL1* and *ASPN*. At 7 days post-surgery, the mRNA corresponding to the *FOSL1* gene showed a significant FC increase of about 1.7 for the Li^+^ group as compared with Ctrl (p = 0.012, Fig. 4A), whereas the *ASPN* gene displayed a significantly increased FC of around 3.5 at 28 days for the Li^+^ group compared to Ctrl (p = 0.03, Fig. 4B).

**Table 8:** Validation of the microarray data by qPCR.

Gene symbol	Gene description	Affymetrix FC Ctrl day 28 vs. 7 (n = 6)	qPCR FC Ctrl day 28 vs. 7 (n = 8)	Affymetrix FC Li^+^ day 28 vs. 7 (n = 6)	qPCR FC Li^+^ day 28 vs. 7 (n = 8)
Prss34	Protease, serine, 34	3.42*	15.97**	3.45*	22.75**
Prkca	Protein kinase C, alpha	1.41***	5.78*	1.29**	3.00***
Lrp5	Low density lipoprotein receptor-related protein 5	-		−1.26*	1.33
Fosl1	Fos-like antigen 1	−1.26***	1.26	−1.30**	−2.26**
Fzd2	Frizzled family receptor 2	−1.68**	−3.40**	−1.66**	−2.47***
Dkk1	Dickkopf 1 homolog (Xenopus laevis)	-		−1.91*	−1.18
Sfrp5	Secreted frizzled-related protein 5	−2.08**	−1.01	-	
Acan	Aggrecan	−2.77**	−2.49*	−2.31**	−1.48
Aspn	Asporin	−2.91*	−10.42***	−3.38**	−2.19*
A2m	Alpha-2-macroglobulin	−3.19***	−6.19***	−4.01***	−5.79***
Cxcl2	Chemokine (C-X-C motif) ligand 2	−4.76*	−7.67*	-	

Relative gene expression fold change (FC) quantified by qPCR in Li+ or Ctrl peri-implant bone, 28 day vs. 7, postoperatively compared with Affymetrix FC (-  =  undetectable expression). Unpaired Student's t-test or Mann-Whitney test was used for statistical significance analyses (* p<0.05, ** p<0.01, ***p<0.001).

### Verification of β-catenin, FOSL1 and ASPN expression

**Figure 5:**
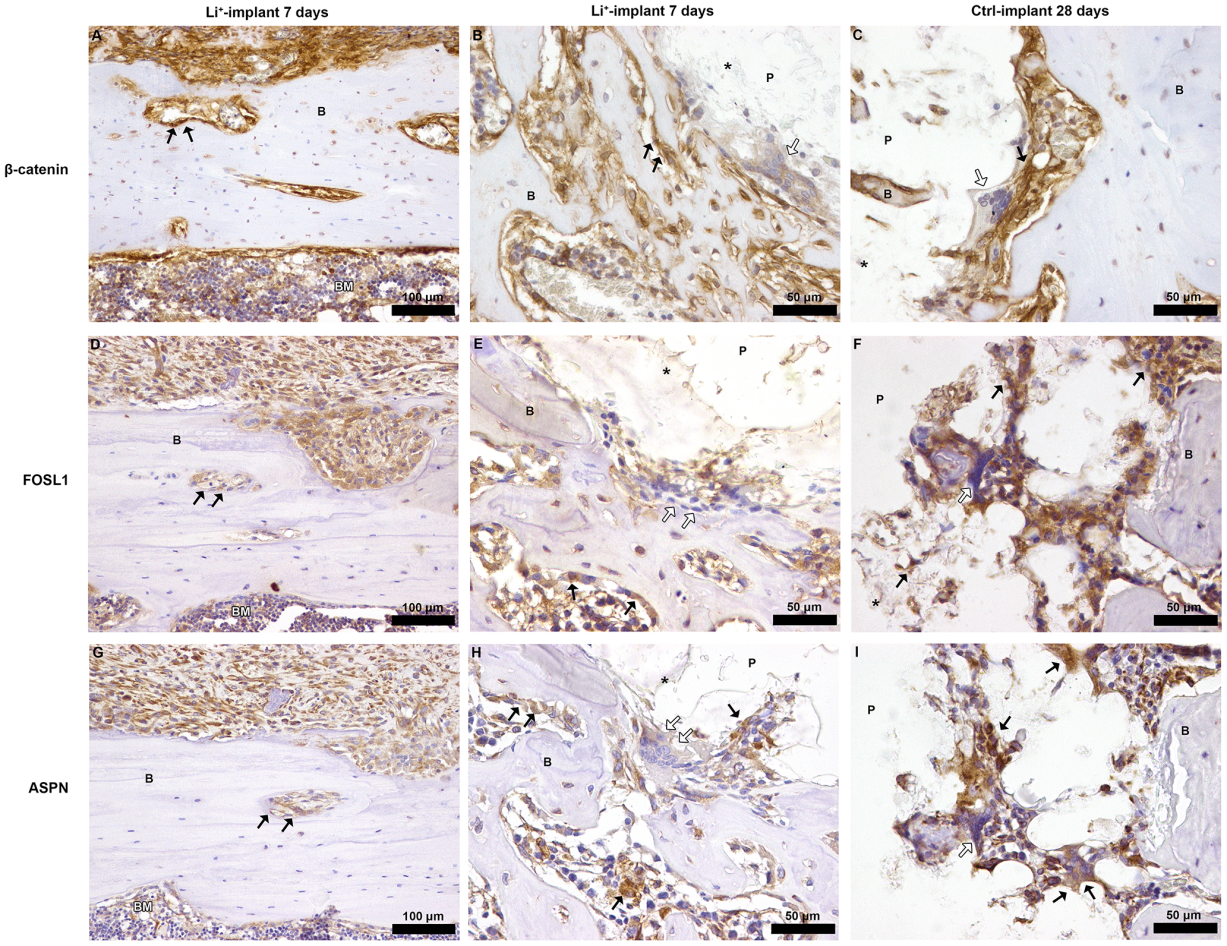
Immunostaning of β-catenin, FOSL1 and ASPN in decalcified bone- implant sections, 7 and 28 days postoperatively. **A.** Li^+^-implant section at 7 days, strong β-catenin immunoreactivity in periosteum, endosteum and within bone marrow cavities in old cortical bone (arrows) was observed. **B.** Li^+^-implant section at 7 days, β-catenin-positive bone-lining cells (black arrows) within bone marrow cavities. Mono- and multinucleated β-catenin-positive cells facing the implant interface were observed, but also less positive multinucleated cells were observed (white arrows). **C.** A similar distribution of β-catenin immunoreactivity was observed 28 days post-surgery in a Ctrl-implant section, with cells migrating into PLGA remnants (black arrows). Occasionally, multinucleated cells with less intense β-catenin staining were found at the bone-implant interface (white arrows). **D.** Li^+^-implant section at 7 days, FOSL1-immunostaining in periosteum, bone depressions and within bone marrow cavities in old cortical bone (arrows). **E.** FOSL1-positive bone-lining cells within bone marrow cavities at 7 days in a Li^+^-implant section (black arrows). Mono- and multinucleated cells positive for FOSL1 migrated into, or were seen in close vicinity of PLGA remnants (black arrows). Some multinucleated cells less positive for FOSL1 were occasionally observed facing the bone-implant interface (white arrows). **F.** Ctrl-implant at 28 days, both mono- and multinucleated FOSL1-positive cells were detected at the bone-implant interface (black arrows). **G.** Li^+^-implant section at 7 days, ASPN immunoreactivity in periosteum, bone depressions and bone marrow cavities (arrows) of the cortical bone. **H.** Li^+^-implant section at 7 days, ASPN-positive osteoblast seams within bone marrow cavities and mesenchymal-like and multinucleated cells located at the PLGA-bone interface (black arrows). Occasional multinucleated cells that displayed lower immunoreactivity were observed at the interface (white arrows). **I.** A similar distribution of ASPN immunoreactivity was detected 28 days post-implantation in a Ctrl-implant section, showing ASPN-positive cells actively migrating into the polymeric remnants (black arrows). All sections (A-I) were counterstained with hematoxylin, control sections with isotype staining were used (data not shown). B  =  bone; P = denotes the location of the (removed) PLGA implant, *  =  indicates the PLGA remnants, BM  =  bone marrow. No qualitative or quantitative histological differences were detected between Li^+^ and Ctrl.

In order to verify the microarray data, the spatial protein expression of β-catenin, FOSL1 and ASPN was studied in peri-implant bone retrieved from Li^+^-containing and Ctrl implants. The proteins were investigated due to their involvement in Wnt signaling and/or increased gene expression by microarray and qPCR. The expression of β-catenin, FOSL1 and ASPN was verified at both time points in bone from both the Li^+^ and Ctrl samples, although no qualitative or quantitative histological differences were observed between the implant groups. Positive immunoreactivity for these proteins was demonstrated in periosteum and bone marrow cavities, as well as on cells situated in the close vicinity of the bone-implant interface (Fig. 5A-I).

### Histomorphometry, histology and immunohistochemistry

**Figure 6:**
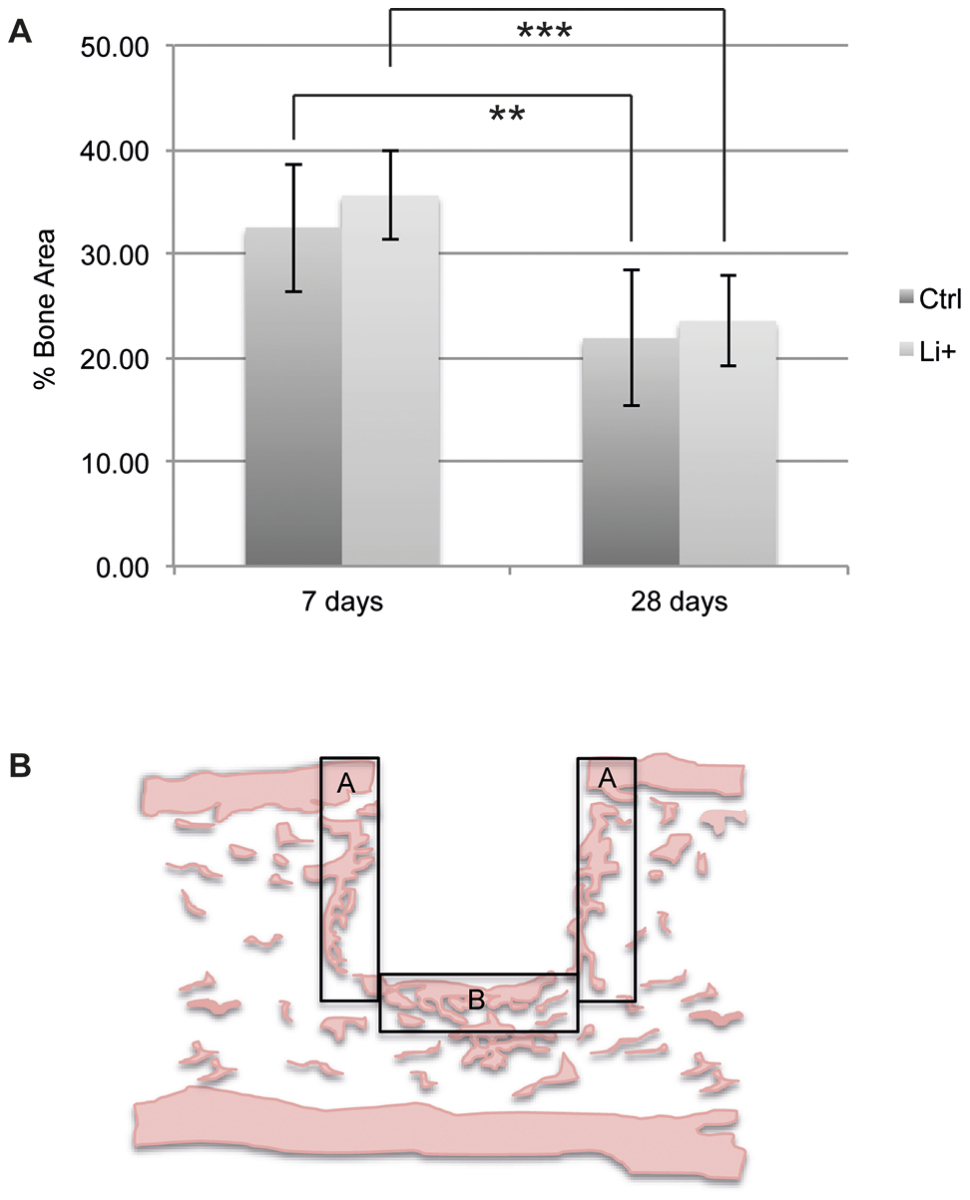
Total bone area around PLGA implants. Histomorphometry. **A.** Total bone area (BA) (%) after 7 and 28 days. The diagram shows mean values and standard deviations. An independent samples T-test was used to compare day 28 vs. 7 for Li^+^ and Ctrl respectively (n = 6–7, * p<0.05, ** p<0.01, ***p<0.001). **B.** Examination areas (A and B) of histological samples.

**Figure 7:**
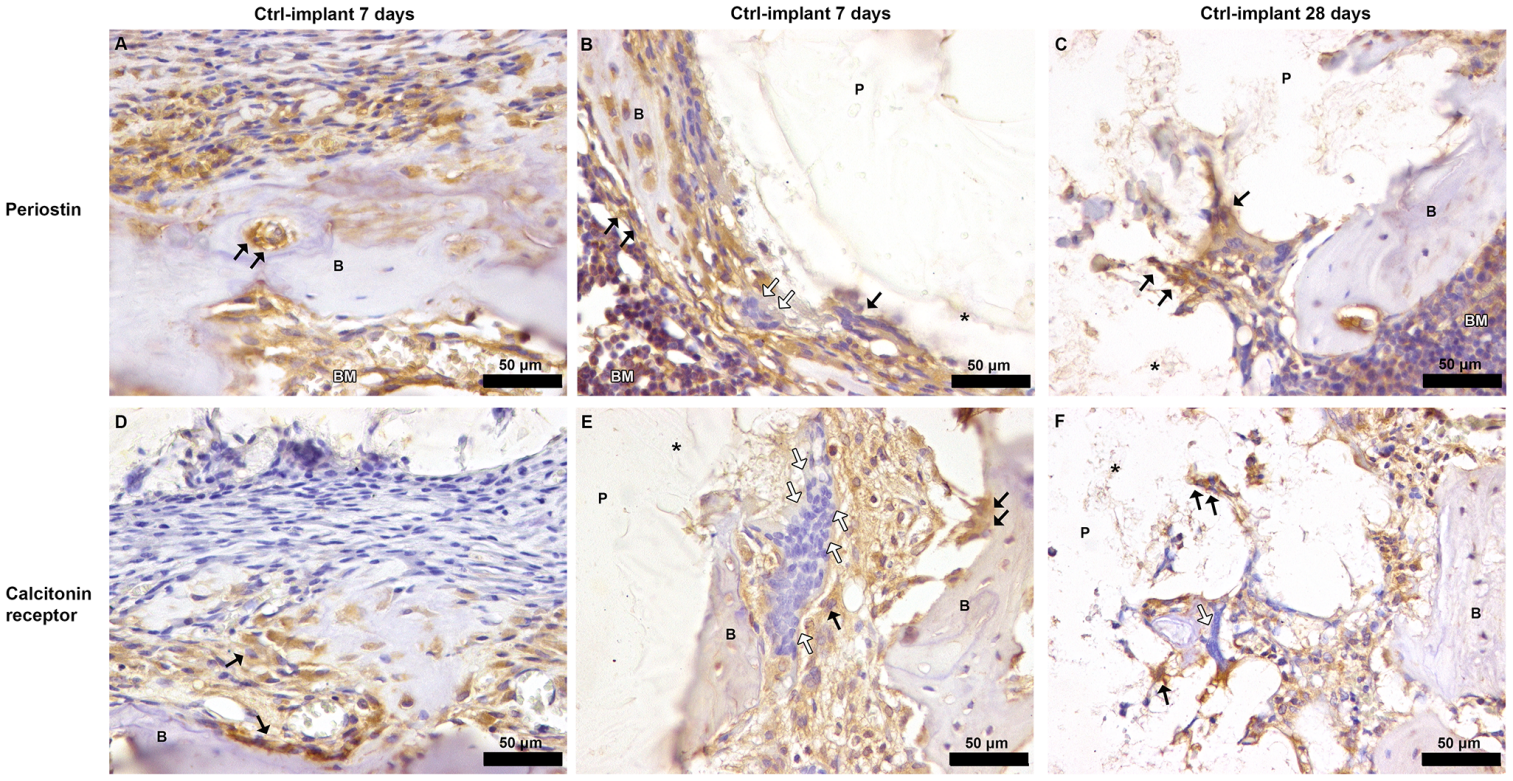
Spatial localization of periostin and calcitonin receptor in decalcified bone-Ctrl implant sections, 7 and 28 days postoperatively. Staining with antibodies against periostin and calcitonin receptors in decalcified paraffin-embedded sections of the bone-implant interface after 7 or 28 days of implantation to rat tibia. **A.** Ctrl-implant section after 7 days, immunoreactivity for periostin was observed over the periosteum and within bone marrow cavities of old cortical bone (arrows). **B.** Periostin-positive cells were distributed throughout the regenerated tissue in Ctrl-implant section 7 days postoperatively. Osteoblast bone lining cells (some indicated by black arrows) were stained strongly for periostin. Immunoreactivity for periostin was observed in mono- and multinucleated cells situated in the close vicinity of the PLGA implant, but occasional less stained multinucleated cells were also observed (white arrows). **C.** A similar distribution of immunohistochemical reaction for periostin was detected after 28 days in a Ctrl-implant section, with cells migrating into the polymeric remnants (arrows). **D.** 7 days postoperatively Ctrl-implant section, immunohistochemical reaction for calcitonin receptor over the inner osteogenic layer of periosteum. **E.** 7 days after implantation Ctrl-implant section, positive staining for the calcitonin receptor was detected on both mono- and multinucleated cells localized preferentially at osteoblast seams and at the bone-implant interface. Osteoclasts and mononuclear precursors stained for the calcitonin receptor are indicated (black arrows). Occasional multinucleated cells negative for calcitonin-receptor immunoreactivity were observed facing the bone-implant interface (white arrows). **F.** A similar distribution of immunohistochemical reaction for calcitonin receptor was detected after 28 days in a Ctrl-implant section, with cells migrating into the polymeric remnants (black arrows). Some multinucleated cells negative for calcitonin receptor are shown (white arrows). All sections (A-F) were counterstained with hematoxylin, control sections with isotype staining was used (data not shown). B  =  bone; P  =  denotes the location of the (removed) PLGA implant, *  =  indicates the PLGA remnants, BM  =  bone marrow. No qualitative or quantitative histological differences were detected between Li^+^ and Ctrl.

Histomorphometry of HE-stained sections revealed no significant differences in bone area (BA) between Li^+^ and Ctrl groups at any time point (Fig. 6A, exam areas see Fig. 6B). On the other hand, a significant decrease in BA over time was demonstrated for both groups; Li^+^ 7 days 35.58%±4.20% vs. 28 days; 23.53%±4.29% (p = 0.0003) and Ctrl 7 days 32.51%±6.15% vs. 28 days 21.89%±6.52% (p = 0.008) (Fig. 6A). The spatial locations of periostin and calcitonin receptors were studied in decalcified paraffin-embedded sections of the tissue defect, 7 and 28 days postoperatively. Periostin-positive cells were distributed throughout the regenerated tissue and osteoblast bone-lining cells were strongly stained for periostin. Positive staining for the calcitonin receptor was observed at 7 days, on both mono- and multinucleated cells localized at osteoblast seams on the bony surfaces facing the bone marrow and at the bone-implant interface. Similar patterns were detected 28 days postoperatively. No qualitative or quantitative histological differences were observed between the implant groups (Fig. 7A-F).

## Discussion

The inclusion of Li^+^ in PLGA was obtained by a hot-melt procedure, resulting in the incorporation of 10% (w/w) Li_2_CO_3_/implant. The SEM and TOF-SIMS imaging data demonstrated how the polymer implant transformed in appearance from a dense to a porous morphology with time. The increased porosity was initiated at the surface of the shaft and spread towards the center. The recognized degradation mechanisms of biodegradable polymers are surface or bulk erosions. Bulk erosion is the dominant mechanism, as water penetrates into the matrix and the degradation occurs in the inner parts of the matrix, leading to an equally distributed porosity. For matrices with dimensions below 7 cm, like our implants, the suggested degradation mechanism is bulk erosion[38]. However, our experimental findings, showing increased porosity close to the surface, indicate that surface erosion contributed largely to the erosion of the device. Furthermore, the *in vitro* Li^+^ release rate was almost constant over time, indicating that the release mechanism follows Case II, i.e. water penetration was the rate-limiting factor and not the drug diffusion out from the device. Inside the PLGA matrix, the highly water-soluble Li_2_CO_3_ was distributed in the shape of particles. When the water front reached the Li_2_CO_3_ particles, these dissolved rapidly and Li^+^ diffused out from the matrix, after which the location of the former Li_2_CO_3_ particles turned into water-filled pores. The rate-limiting step for Li^+^ release was therefore the water penetration rate. Simultaneously, the pore formation observed in SEM and TOF-SIMS was also controlled by water penetration. After 28 days of exposure to buffer, about 50% of the Li^+^-salt content was released, and even though highly perforated the implants still held together. In order to obtain a complete PLGA degradation or a larger amount of released salt, the formulation has to be further optimized. The mode of degradation in bone is also expected to be different from that of a fully submerged implant in PBS buffer, because of the uniqueness of the bone environment. However, assuming an equal *in vitro* and *in vivo* release rate, this means that during 28 days, about 2.3 10^−5^ mol Li^+^ was released to the bone environment around the implants.

The results of the microarray analysis revealed the largest number of significantly differentially regulated genes within each respective implant group over time and the Li^+^-containing implants induced a somewhat higher total number of regulated genes compared with Ctrl. This is in line with a recent study by Thalji *et al*.[28] and illustrate the complex process of intramembranous bone regeneration; the principal healing route around bone-anchored implants[29]. In a process like this, including an initial inflammatory phase followed by osteogenesis, woven bone formation and finally bone remodeling, it is likely that minor alterations induced by the different implants become hidden in the more powerful process of inherent bone healing, growth and remodeling. The bone regeneration presumably also involves a much larger subset of genes than the different implant groups affect themselves. Moreover, the microarrays generate information about several thousands of genes in a single experiment and it is inevitable that genes with low FC disappear within this context. Nevertheless, we cannot rule out the possibility that gene expression differences demonstrating low FC in fact lead to a significant biological outcome when related to sets of the same genes and participate in the same biological process, as demonstrated in earlier work[39]. When using the specific search criteria (FC≥1.5 or ≤−1.5; p<0.05), a larger number of genes were downregulated rather than upregulated over time. The result demonstrates that the process of bone healing is powerful in the early phases and decreases over time. The result was expected and is in accordance with the results presented by others[29], [30], demonstrating that the early phases of osseointegration (days 4–14) involve a large change in the expression of genes related to proliferation, while immuno/inflammatory responses were most prevalent at day 4 and skeletogenesis-associated genes dominated in the later phase. In contrast to the above, Kojima *et al*. demonstrated that more genes were upregulated during the later time point of healing[32]. When considering the functional clustering with respect to biological functions as a whole, no major overall differences between Li^+^-containing samples and Ctrls were revealed. Both implant groups demonstrated clusters relating to the extracellular matrix organization, inflammatory response, cell migration, blood vessel development and wound healing. These were significantly downregulated over time, as expected. Furthermore, both implant groups showed clusters related to neuron development, differentiation axongenesis and axon guidance. Interestingly, these clusters contained a larger number of genes (Li^+^ 36; Ctrl 25) than skeletogenesis-related genes (Li^+^ 8; Ctrl 12), a surprising finding. Similar results were recently published by Ivanovski *et al*.[30], who demonstrated an over-expression of neurogenesis-associated genes at day 14. These findings indicate the formation of neurogenic tissue during the osseointegration process. Apart from the regeneration of nerve fibers, the role of the neurogenesis-associated genes during osseointegration is not fully understood. However, few studies have examined a regulatory connection between the central nervous system and bone remodeling and it is known that leptin regulates bone formation via the central nervous system[40], [41]. However, this requires far more investigation. Further, the Ctrl specimens demonstrated clusters related to the skeletal system, bone development and ossification. The Li^+^-containing implants also demonstrated clusters related to the skeletal system, but in contrast to the Ctrl, also cartilage development and condensation. Although not supported by the present histological observations, this finding might suggest a more endochondral-like peri-implant healing around Li^+^−containing implants and diverges from the principal route of intramembranous bone regeneration. Only 1 annotation cluster, related to the regulation of cell activation, was significantly upregulated over time for the Ctrl group, whereas 2 annotation clusters (related to hemostasis, wounding, regulation of body fluids and cell activation) were significantly upregulated by the Li^+^ group.

The KEGG pathway analysis revealed 6 pathways that were significantly downregulated over time (day 28 vs. 7) for the Ctrl implants. They included focal adhesion, ECM-receptor interaction, arginin and proline metabolisms and axon guidance. In another study, these pathways were upregulated during the early phase of intramembranous bone regeneration[29], which is in line with our findings. Nine pathways were significantly downregulated over time for the Li^+^ group. In addition to focal adhesion, ECM-receptor interaction, arginin and proline metabolism and axon guidance, pathways related to cancer, involving the Wnt signaling pathway, were also downregulated over time. For the Ctrl group, no pathways were significantly upregulated over time, while the Li^+^ group showed 2 pathways as significantly increased at day 28. This included the hematopoetic cell lineage, which Wise *et al*.[29] claimed was downregulated in the early bone healing phase, and is in line with our result. Due to the above-mentioned pathway analysis indicating that the Wnt signaling pathway was affected over time by Li^+^, specific Wnt-related genes were searched for in the KEGG database. This revealed an increased number of affected Wnt-related genes in the context of Li^+^. Furthermore, according to the KEGG database, 98 genes are associated with the Wnt signaling pathway and our results suggest that 4–9% of the Wnt signaling genes were specifically regulated by Li^+^ (at each time point), while approximately 34% was regulated in the Li^+^ group and 22% in the Ctrl group over time. This is regarded as a considerable proportion of affected genes within the same biological process. The Wnt-specific genes showed an overall low FC. However and as discussed above, the Wnt cascade is a well-conserved, essential pathway in osteogenesis and it is recognized that even subtle changes in amplitude and the duration of numerous Wnt-related markers might regulate the entire pathway[9]. Moreover, a low FC may therefore result in a significant biological outcome and this is regarded as a valuable data outcome from the present study. Taken together, this also illustrates the importance of small alterations in gene expression. Both activators and inhibitors of the Wnt pathway were significantly affected over time. β-catenin is a well-documented key mediator of the Wnt signaling pathway[42], [43] and, interestingly, the β-catenin interaction protein, a negative regulator of the Wnt signaling pathway[44], was downregulated for Li^+^ compared with Ctrl, 7 days post-surgery. Further, in our study, β-catenin was downregulated over time by Li^+^, but it was not affected in the Ctrl group, indicating an increased activity in the Wnt signaling pathway by Li^+^ during the early stages of osseointegration and reduced activity in later stages. Our result is also in line with earlier studies demonstrating enhanced Wnt signaling during the early phase of bone regeneration[29], [30]. Other signs of an affected Wnt signaling pathway in bone around Li^+^ implants was demonstrated by the significant increase in the gene expression of *FOSL1* at 7 days post-implantation, compared with Ctrl. FOSL1, also called FRA-1, which is part of the transcription factor, activator protein-1 (AP-1), is a downstream target gene of canonical Wnt signaling[45], thus demonstrating an active signaling pathway around Li^+^ containing specimens. FRA-1 positively regulates the bone-forming activity of osteoblasts and is active during osteoclast differentiation. It has been suggested that ectopic expression induces bone formation and osteosclerosis[46], [47]. Heo *et al*. reported an increase in *FRA-1* gene expression in cells stimulated with Li^+^ [48], a result supported by our data. Moreover, we have previously demonstrated a significant increase in this gene expression in osteoarthritic (OA) cartilage[49]. This is important, as an increase in subchondral plate thickness is a characteristic of OA pathogenesis and underlines the essential function performed by FOSL1 in bone regeneration.

Li^+^-containing implants displayed at 28 days post-surgery a significant upregulation in *ASPN* gene expression compared with Ctrls. ASPN is a member of the small leucine-rich repeat proteoglycan (SLRP) family (subgroup of the leucine-rich repeat (LRR) superfamily of extracellular matrix proteins) and is recognized as a negative regulator of both early- and late-stage chondrogenesis, with increased expression in OA, and is expressed in periosteum during development[50]. Kalamajski *et al.* have shown that ASPN binds calcium and collagen type 1 and is suggested to be involved in osteoblast-driven biomineralization. An increase in the gene expression of *Osteorix* and *RUNX2* was also reported in relation to ASPN[51]. It has also been suggested that the active canonical Wnt pathway inhibits chondrogenesis and is implicated in the pathogenesis of OA[52], [53]. Moreover, a recent publication reported that sclerostin, an extracellular inhibitor of the canonical Wnt pathway that is primary expressed by osteocytes and binds to LRP5/6[54], [55], interacts with ASPN[56]. We speculate that the significant increase in *ASPN* gene expression around Li^+^ implants at 28 days post-implantation is somehow related to an active Wnt signaling caused by the locally released Li^+^, or suggests a connection between Wnt signaling and ASPN expression. The distribution of β-catenin, FOSL1 and ASPN in bone was investigated using IHC. Unfortunately, some of the sections were of inferior sample quality due to artifacts created during sample preparation and it was therefore not possible to perform meaningful comparisons at the actual interface between PLGA and bone. However, positive stainings for these markers were shown in periosteum and bone marrow cavities. Immunoreactivity for β-catenin, FOSL and ASPN was also observed in cells actively migrating into the polymeric remnants, thus demonstrating that the expressions of these markers were located within tissues with osteoblast progenitor cells and in areas with active woven bone formation. Moreover, multinucleated cells at the bone-implant interface were occasionally negatively stained for these markers. However, in order fully to investigate potential differences in protein expression in the vicinity of the implants, additional techniques for quantitative analyses will be needed (e.g. proteomics or western blot) and the precise role of these genes in the modulation of peri-implant healing requires further examination. In addition to the distribution and expression of β-catenin, FOSL1 and ASPN, the histomorphometric analysis demonstrated a significant decrease in bone area for both implant types over time. This may be a result of the mild inflammatory response that could be induced by small PLGA particles (de-attached during the implantation process) and/or degradation products over time[57], [58], although further evaluation is needed. Activated macrophages and a foreign-body reaction towards biomaterials is known to secrete a wide range of cytokines[59] that are regarded as osteoclastogenic[60], thereby possibly affecting bone resorption in the vicinity of implants. Further, both osteoblastic activity and the number of macrophages and giant cells have been studied in the context of PLGA[23]. These results were supported by the present histological evaluation showing the occasional presence of multinucleated giant cells within the space between bone and PLGA remnants. In order to further investigate the spatial localization of different cell types in the close vicinity of the polymeric implants, the expression of periostin, involved in the early stages of osteoblast differentiation[61], and the calcitonin receptor, expressed on osteoclasts[62], were visualized by IHC. Positive stainings were observed at both time points for both markers, although no qualitative or quantitative evaluation over time was possible (due to inferior sample quality). Periostin-positive cells were distributed throughout the regenerated tissue and multinucleated cells positive for the calcitonin receptor were observed at the bone-implant interface. Occasionally, periostin- and calcitonin receptor-negative multinucleated giant cells were observed in the close vicinity of the polymer implants. The absence of these and the abovementioned markers, as well as the localization of these cells, likely indicate foreign-body-reaction-associated giant cells and/or alternatively inflammation-associated cells. Some of the most downregulated inflammation related genes over time for both groups revealed in the microarray analysis were *A2M* and *CCL7*, whereas *CXCL2* was downregulated only in the Ctrl group. A2M is a protease inhibitor known to exert a key role in the immune system although its mechanisms are not completely understood. It is associated with tissue destruction in periodontitis and is suggested to inhibit inflammation and to suppress proliferation of microorganisms[63]. A2M is also implicated in cartilage degradation, it is known to interact with the osteogenic growth peptide, osteoprotegerin (OPG), and suggested to inhibit BMP-1[64]. Analysis of proteins bound to different biomaterial surfaces recently identified A2M resorbed on PLGA [65]. Earlier studies with biomaterials with this pre-adsorbed protein facilitate macrophage fusion and formation of foreign body giant cells[66]. The progression of inflammation and foreign body response involves the migration of monocytes and macrophages to the site of implantation, which is guided by chemokines and other chemoattractants. The *CCL7* gene encodes the monocyte chemotactic protein 3 (MCP-3), a chemokine implicated as a key mediator of pro-inflammatory pathways, recognized to activate leukocytes and to attract monocytes/macrophages during inflammation[59]. CCL7 has further been identified as a MSC homing factor in cardiac tissue[67], a function of the chemokine that Shinohara *et al*. also recently have suggested to participate in bone repair[68]. CXCL2 (also known as MIP-2) is involved in recruitment of monocytes and macrophages into inflamed tissues by bone marrow MSCs[69], and both mast cells and macrophages are known to secrete this chemokine that also acts as a neutrophil chemoattractant during tissue inflammation[70]. Chemokines are believed to play important functions in several aspects of bone metabolism including for instance recruitment of leukocytes and osteoclast maturation, and recently CXCL2 was observed to be upregulated at fracture sites of diabetic mice[71]. However, despite that the functional cluster analysis demonstrated a slight change in enrichment score between Li^+^ and Ctrl implants with respect to the inflammatory response and the above-mentioned genes, no qualitative histological differences were detected and further evaluation is required.

Taken together, our results demonstrated a large number of genes as regulated over time, and with a large subset of Wnt-related markers involved. Although the significantly increased expression of the Wnt-associated genes, *FOSL1* and *ASPN*, was related to Li^+^, no obvious effect of an activated Wnt pathway in the context of increased bone regeneration was observed. We want to stress here that this observation was made for a bioresorbable material, PLGA with and without added Li^+^, and may be different for different biomaterials. On the other hand and very interestingly, both implant groups showed a significant decrease in bone area over time. This might reflect a sustained mild inflammatory response caused by PLGA degradation products. Our data provide a basis for further studies of the function of Wnt-related genes in peri-implant healing around polymeric implants and the modulatory effect of Li^+^, although further progress will be required to optimize Li^+^ administration for a local therapeutic effect.

## Conclusions

In the present study, Li^+^ was successfully released from PLGA implants out to surrounding bone in a sustained release fashion and the gene expression profile during peri-implant healing in a rat tibia model was investigated. Microarray analysis revealed a large number of significantly differentially regulated genes within the 2 implant groups (PLGA with/without Li^+^) over time. The annotation cluster analysis demonstrated, for both implant groups, skeletal system morphogenesis/development and, surprisingly, the Li^+^-containing implants were also related to cartilage development and condensation as well as to the Wnt signaling pathway. Li^+^ appears not to be a strong inducer of early bone growth around PLGA implants although the qPCR analysis demonstrated that Li^+^ activated the Wnt signaling pathway at 7 days post-surgery, as demonstrated by a significant increase in *FOSL1* expression. A possible impact on the Wnt signaling pathway via *ASPN* expression was observed at 28 days post-surgery. The decrease in bone area that was observed around both implant groups may to some extent be explained by the presence of multinucleated cells, possibly caused by the polymeric degradation products. The collected data indicate that Li^+^ is a mild bone growth modulator in the context of bone-anchored implants and the Wnt signaling pathway when administered locally in the present dose and during the time frame of the study, up to 28 days.

## Supporting Information

Table S1 **List of all differentially regulated genes between Li^+^ day 7 vs. Ctrl (p< 0.05, no specific fold change (FC) limit).** Genes with a positive FC indicate genes upregulated in Li^+^ and negative FC values indicates downregulated genes.
(XLSX)

Table S2 **List of all differentially regulated genes between Li^+^ day 28 vs. Ctrl (p<0.05, no specific fold change (FC) limit).** Genes with a positive FC indicate genes upregulated in Li^+^ and negative FC values indicates downregulated genes.
(XLSX)

Table S3 **List of all differentially regulated genes between Ctrl day 28 vs. 7 (p<0.05, no specific fold change (FC) limit).** Genes with a positive FC indicate genes upregulated at 28 days and negative FC values indicates downregulated genes.
(XLSX)

Table S4 **List of all differentially regulated genes between Li^+^ day 28 vs. 7 (p<0.05, no specific fold change (FC) limit).** Genes with a positive FC indicate genes upregulated at 28 days and negative FC values indicates downregulated genes.
(XLSX)

Table S5 **Top 10 results of functional annotation clustering of genes downregulated in Li^+^ day 28 vs. 7.** Enrichm. Score: the geometric mean (in -log scale) of member's p-values of the corresponding annotation cluster. Genes: total number of different genes within a functional annotation cluster. In parenthesis: number of genes and fold enrichment of the functional term. Only genes that displayed P<0.05, FC≥1.5 or ≤−1.5 were used as input.
(XLSX)

Table S6 **Results of functional annotation clustering of genes upregulated in Li^+^ day 28 vs. 7.** Enrichm. Score: the geometric mean (in -log scale) of member's p-values of the corresponding annotation cluster. Genes: total number of different genes within a functional annotation cluster. In parenthesis: number of genes and fold enrichment of the functional term. Only genes that displayed P<0.05, FC≥1.5 or ≤−1.5 were used as input.
(XLSX)

Table S7 **Top 10 results of functional annotation clustering of genes downregulated in Ctrl day 28 vs. 7.** Enrichm. Score: the geometric mean (in -log scale) of member's p-values of the corresponding annotation cluster. Genes: total number of different genes within a functional annotation cluster. In parenthesis: number of genes and fold enrichment of the functional term. Only genes that displayed P<0.05, FC≥1.5 or ≤−1.5 were used as input.
(XLSX)

Table S8 **Results of functional annotation clustering of genes upregulated in Ctrl day 28 vs. 7.** Enrichm. Score: the geometric mean (in -log scale) of member's p-values of the corresponding annotation cluster. Genes: total number of different genes within a functional annotation cluster. In parenthesis: number of genes and fold enrichment of the functional term. Only genes that displayed P<0.05, FC≥1.5 or ≤−1.5 were used as input list.
(XLSX)

Table S9 **List of all differentially regulated Wnt genes between Li^+^ day 7 vs. Ctrl.** P<0.05, no specific fold change (FC) limit. Genes with a positive FC indicate genes upregulated in Li^+^ and negative FC values indicate downregulated genes.
(XLSX)

Table S10 **List of all differentially regulated Wnt genes between Li^+^ day 28 vs. Ctrl.** P<0.05, no specific fold change (FC) limit. Genes with a positive FC indicate genes upregulated in Li^+^ and negative FC values indicate downregulated genes.
(XLSX)

Table S11 **List of all differentially regulated Wnt genes between Li+ day 28 vs. 7.** P<0.05, no specific fold change (FC) limit. Genes with a positive FC indicate genes upregulated at 28 days and negative FC values indicate downregulated genes.
(XLSX)

Table S12 **List of all differentially regulated Wnt genes between Ctrl day 28 vs. 7.** P<0.05, no specific fold change (FC) limit. Genes with a positive FC indicate genes upregulated at 28 days and negative FC values indicate downregulated genes.
(XLSX)
